# Exploiting Genomic Knowledge in Optimising Molecular Breeding Programmes: Algorithms from Evolutionary Computing

**DOI:** 10.1371/journal.pone.0048862

**Published:** 2012-11-21

**Authors:** Steve O'Hagan, Joshua Knowles, Douglas B. Kell

**Affiliations:** 1 School of Chemistry, The University of Manchester, Manchester, United Kingdom; 2 The Manchester Institute of Biotechnology, The University of Manchester, Manchester, United Kingdom; 3 School of Computer Science, The University of Manchester, Manchester, United Kingdom; University of Vermont, United States of America

## Abstract

Comparatively few studies have addressed directly the question of quantifying the benefits to be had from using molecular genetic markers in experimental breeding programmes (e.g. for improved crops and livestock), nor the question of which organisms should be mated with each other to best effect. We argue that this requires *in silico* modelling, an approach for which there is a large literature in the field of evolutionary computation (EC), but which has not really been applied in this way to experimental breeding programmes. EC seeks to optimise measurable outcomes (phenotypic fitnesses) by optimising *in silico* the mutation, recombination and selection regimes that are used. We review some of the approaches from EC, and compare experimentally, using a biologically relevant *in silico* landscape, some algorithms that have knowledge of where they are in the (genotypic) search space (G-algorithms) with some (albeit well-tuned ones) that do not (F-algorithms). For the present kinds of landscapes, F- and G-algorithms were broadly comparable in quality and effectiveness, although we recognise that the G-algorithms were not equipped with any ‘prior knowledge’ of epistatic pathway interactions. This use of algorithms based on machine learning has important implications for the optimisation of experimental breeding programmes in the post-genomic era when we shall potentially have access to the full genome sequence of every organism in a breeding population. The non-proprietary code that we have used is made freely available (via Supplementary information).

## Introduction

A common circumstance in biotechnology [1] is that we have an entity (be it a protein, nucleic acid or organism) that we wish to improve for some specific purposes, and this is largely done by genetic means, involving either the mutation of a single parent entity or (if mating is possible) by means that additionally involve genetic recombination. Of course, observations of the success of experimental breeding (or ‘directed evolution’) contributed significantly to Darwin's thinking, even before we knew anything about the existence of genes, and many examples (e.g. [2]–[5]) show the huge variation in phenotype achievable in inter-breeding populations. The usual metaphor here, due to Sewall Wright [6], is that of a ‘fitness landscape’ that relates genotype (i.e. sequence) to fitness, and that involves moving around this landscape by mutation and recombination while seeking to improve the fitness(es) of our offspring en route to some variety that is highly improved relative to the starting position.

We note too that we are usually interested in optimising for multiple traits, which may be largely independent of or at best only partially linked to each other (e.g. disease resistance in a crop plant is essentially independent of the metabolic processes governing primary yield, but would potentially contribute to overall yield, and we might select for larger roots [7], [8] separately from the agronomic benefits such as drought tolerance that they might bring). This makes our searches for improved strains a multi-objective optimisation problem [9]. Of course plant and animal breeding holds considerable potential for improving food security, a topic of substantial current interest [10]–[17].

**Table 1:** Some evolutionary algorithms: F-Algorithms.

ALGORITHM	COMMENTS	REFERENCES
Breeder genetic algorithm	Basic evolutionary algorithm using truncation selection	[74], [148]
Phenotypic niching with fitness sharing	The reproductive opportunities of individuals are shared amongst members of a niche. A niche is defined by a neighbourhood in phenotype-space, i.e. as a vector of attributes or traits. The scheme seeks to preserve diversity.	[130], [149]
Deterministic crowding	Crowding is a reproduction scheme in which individuals are forced to replace individuals in the population that are most like them. In deterministic crowding this is achieved without inspection of the genotype; offspring merely replace their parents (depending on the relative fitness of the parents and offspring). Preserves diversity.	[150], [151]
Local selection; local breeding; local mating; spatially structured populations	The population is given some spatial structure (usually independently of fitness), and mating is allowed to occur only between neighbours in this structure. Similarly, offspring replace low-fitness individual(s) within their own neighbourhood. Preserves diversity.	[152]
Island model GAs (Alba and Tomassini)	Several populations evolve on separate islands using locally panmictic mating. There is limited but occasional migration from one island to the other. Preserves diversity.	[54], [153]–[155]
Landscape state machine tuning of directed evolution (LSM-DE)	In this technique, choices for mutation rate, population size, selection pressure and other evolutionary parameters are based on some prior sampling – and subsequent modelling – of the fitness landscape (or one believed to have similar topological features). Tunes the search algorithm specifically to the problem.	[90], [156], [157]
Fitness uniform selection scheme (FUSS)	FUSS is a selection scheme that preserves phenotypic diversity.	[158], [159]
Hybrid local search or memetic algorithms	Evolution scheme in which selected individuals (usually fitter ones) in each generation are improved by performing a fitness-directed walk on the landscape. Improves exploitation of fit individuals, driving them towards optima. May not be feasible for some types of Directed Evolution experiment. These may not perform well when large populations but small numbers of generations are available (since the adaptive walks necessarily take the equivalent of several breeding generations to complete.)	[160], [161]
Statistical Racing	Several evolutions, each with different parameters controlling selection pressure, mutation rates, and so on, are run simultaneously. At intervals, any evolution that is performing statistically significantly worse is dropped and its resources are allocated equally to the others. Expensive but effective.	[162], [163]
Self-tuning evolutionary algorithms	Mutation rates, selection pressure, rates of recombination are controlled during evolution. These may be changed deterministically according to a schedule; changed according to some rules based on the progress being made; or actually evolved by making the parameters themselves subject to selection and variation.	[60]

The issue, then as now, is that mostly we carry out breeding experiments ‘in the dark’ genetically, and though we may seek to combine complementary traits from individual parents (whether phenotypically or via Quantitative trait loci (QTLs)), the number of experiments we can typically carry out in a generation is tiny relative to the number of possible matings (given the size of the parental populations available) – if there are m and f possible parents of each gender the number is obviously mf. Note too the gigantic (genetic) sequence space: a microarray of just the set of 30mers, made using spots of just 5 μm diameter, would cover 29 km^2^ [18]! This begs the question of how we optimise the breedings we choose to perform, i.e. which parents we select to mate with each other, and which offspring we select for further breeding. It might be assumed that it would simply be best to breed from the fittest parents obtained in the previous generation, but this is well known not to be the case, since (i) the landscapes are both complex and epistatic (the allele in one location affects what is optimal in another location), and (ii) most mutations tend to be deleterious and would therefore be selected out, even if it was in fact necessary to retain them to get to a fitter part of the landscape. In one computational example [19], more than one third of the non-neutral mutations (steps) in an evolutionary programme that were productive overall were via entities with fitness lower, sometimes much lower, than that of their parents. The intrinsic irreversibility of evolution [20] also makes this a significant issue. The high level of epistasis necessarily contingent upon the organisation of gene products into pathways is probably a major contributor to the comparative ineffectiveness of genome-wide association studies that look solely at individual genes [21]–[24], as well as the simple fact that most genes individually contribute very little to the fitness of complex traits overall [23], [25]–[27], albeit that the right combinations of just a few can indeed do so [28].

**Table 2:** Some evolutionary algorithms: G-algorithms.

ALGORITHM	COMMENTS	REFERENCES
Niching by genotypic fitness sharing	Fitness (reproductive opportunity) of individuals is shared amongst members of the same genotypic niche. Maintains diversity.	[130], [149]
Learnable Evolution Model (LEM)	Uses classifiers to learn the genotypic basis of fitness during an evolutionary run; the inferred basis is used to alter selection to favour those with high predicted fitness, and disfavour those inferred to be deleterious.	[131], [164]
Metamodel-assisted EAs	A regression model relating fitness to genotype is learned during evolution. The model is used to filter offspring individuals before they are evaluated (if their predicted fitness is low).	[78]–[81]
Efficient Global Optimisation (EGO, ParEGO)	A regression model of Gaussian process type is used to relate fitness to genotype (based on a sparse initial sampling of individuals). The model is globally searched to find the individual with the best “expected improvement” in fitness. This individual is then evaluated and used to update the model, and the process iterated.	[83], [87], [165]

**Table 3:** Parameter settings used in the *F* and *G* algorithms.

Algorithm	Type	Key parameters
Breeder	*F*	λ = 1000; *p_m_* = 1/*N*
Standard GA	*F*	λ = 1000; *p_m_* = 1/*N*; *p_c_* = 0.7; *t_size_* = 10
Local mating	*F*	λ = 1000; *p_m_* = 1/*N*; *p_c_* = 0.7; *t_size_* = 10
Niching	*G*	λ = 1000; *p_m_* = 1/*N*; *p_c_* = 0.7; *t_size_* = 10; *niche radius* = dynamic with target niche count, *T_q_* = 5
EARL1	*G*	λ = 1000; *p_m_* = 1/*N*; *p_c_* = 0.7; *t_size_* = 10
EARL2	*G*	λ = 1000; *p_m_* = 1/*N*; *p_c_* = 0.7; *t_size_* = 10

**Table 4:** Tuneable Algorithm Parameters.

Computation Parameter	Corresponding Breeding Scenario
Population Size, P	Size of experimental population
Mutation Rate	Radiation dose
Cross-over rate	Self-pollination vs Cross-pollination?
Tournament size	Could be done directly via statistical sampling pool used to select breeders.
Niche Radius/Target No. of Niches	Could be done directly by statistical methods of measuring similarity.

While proteins link to pathways, and the enzymes encoding them may be on different chromosomes, in our discussions we essentially assume that we are dealing with one chromosome, as the main analysis of genotype-phenotype mapping cares little [29], [30]. This may lead to a minor issue with respect to the effects or effectiveness of recombination, but the frequency of recombination per meiotic event is not in fact very great in most natural populations [31]–[35] and we shall ignore this.

**Figure 1:**
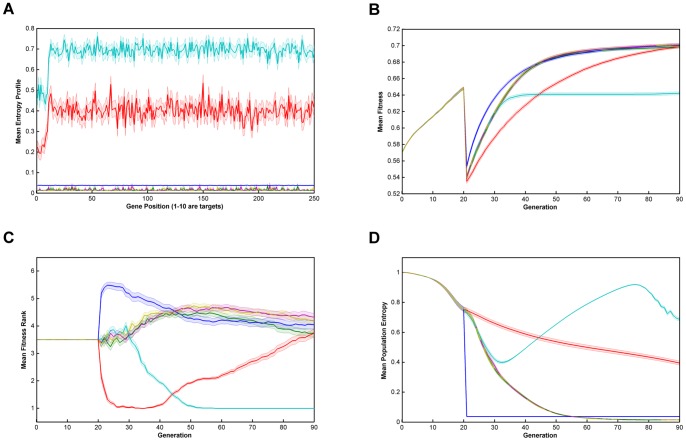
Relative performance of the six algorithms on the modified *NK*-landscapes where λ* = *10000, N = 250, K = 5, r = 10 & burn-in = 20. Note that there a discontinuity in fitness where the algorithm switches from ‘global fitness’, F, to trait fitness, F'. **Key**: **A**. Mean Entropy Profile at Final Generation; **B**. Mean Fitness; **C**. Mean Fitness Rank; **D**. Mean Entropy; —— Breeder; —— Standard GA; —— Local Mating; —— Niching; —— EARL1; EARL2; Error bands are ±1 standard error  = ± stdev/√n.

**Figure 2:**
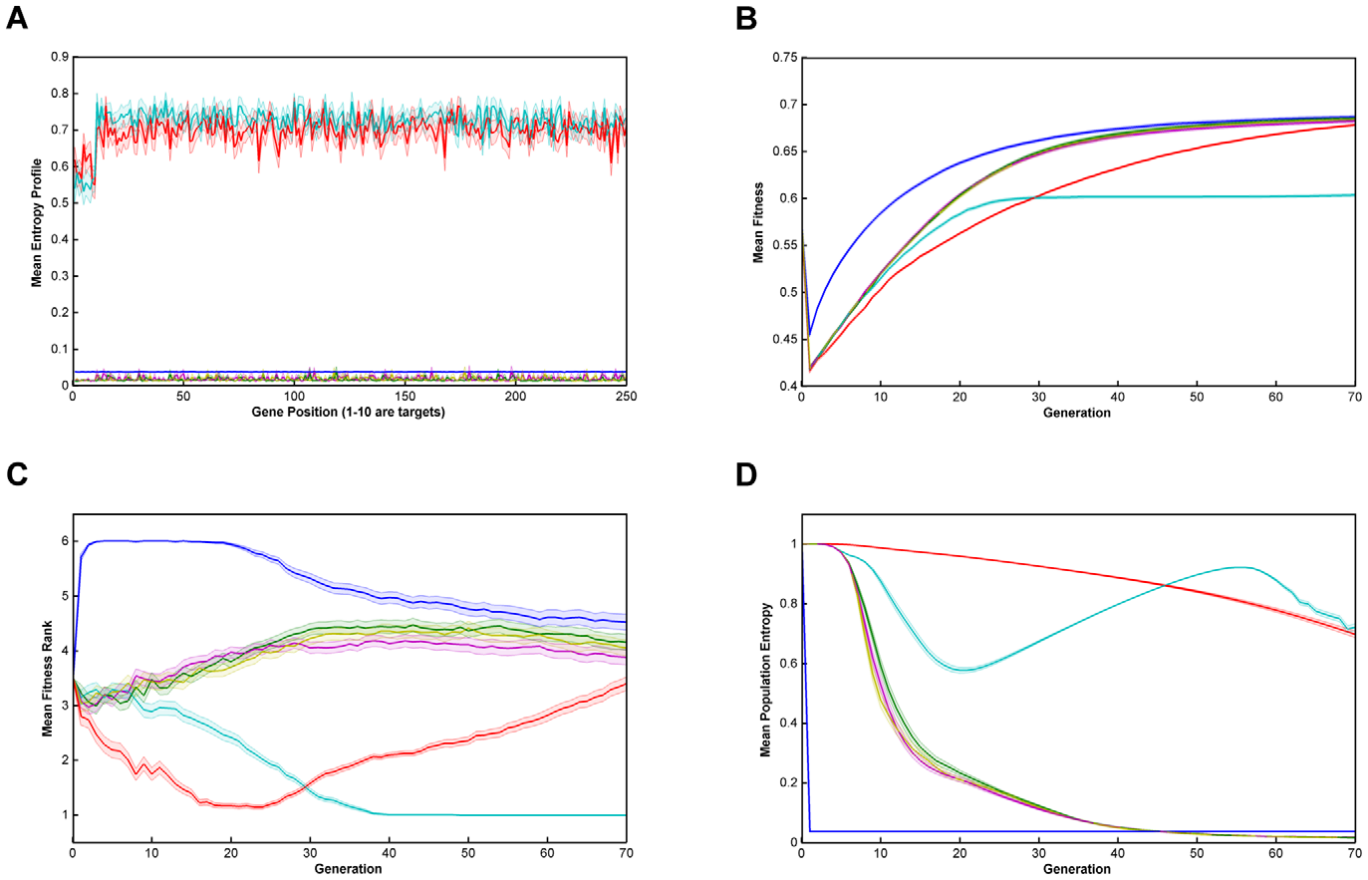
Relative performance of the six algorithms on the modified *NK*-landscapes where λ* = *10000, N = 250, K = 5, r = 10 & no burn-in. **Key**: **A**. Mean Entropy Profile at Final Generation; **B**. Mean Fitness; **C**. Mean Fitness Rank; **D**. Mean Entropy; —— Breeder; —— Standard GA; —— Local Mating; —— Niching; —— EARL1; EARL2; Error bands are ±1 standard error  = ± stdev/√n.

**Figure 3:**
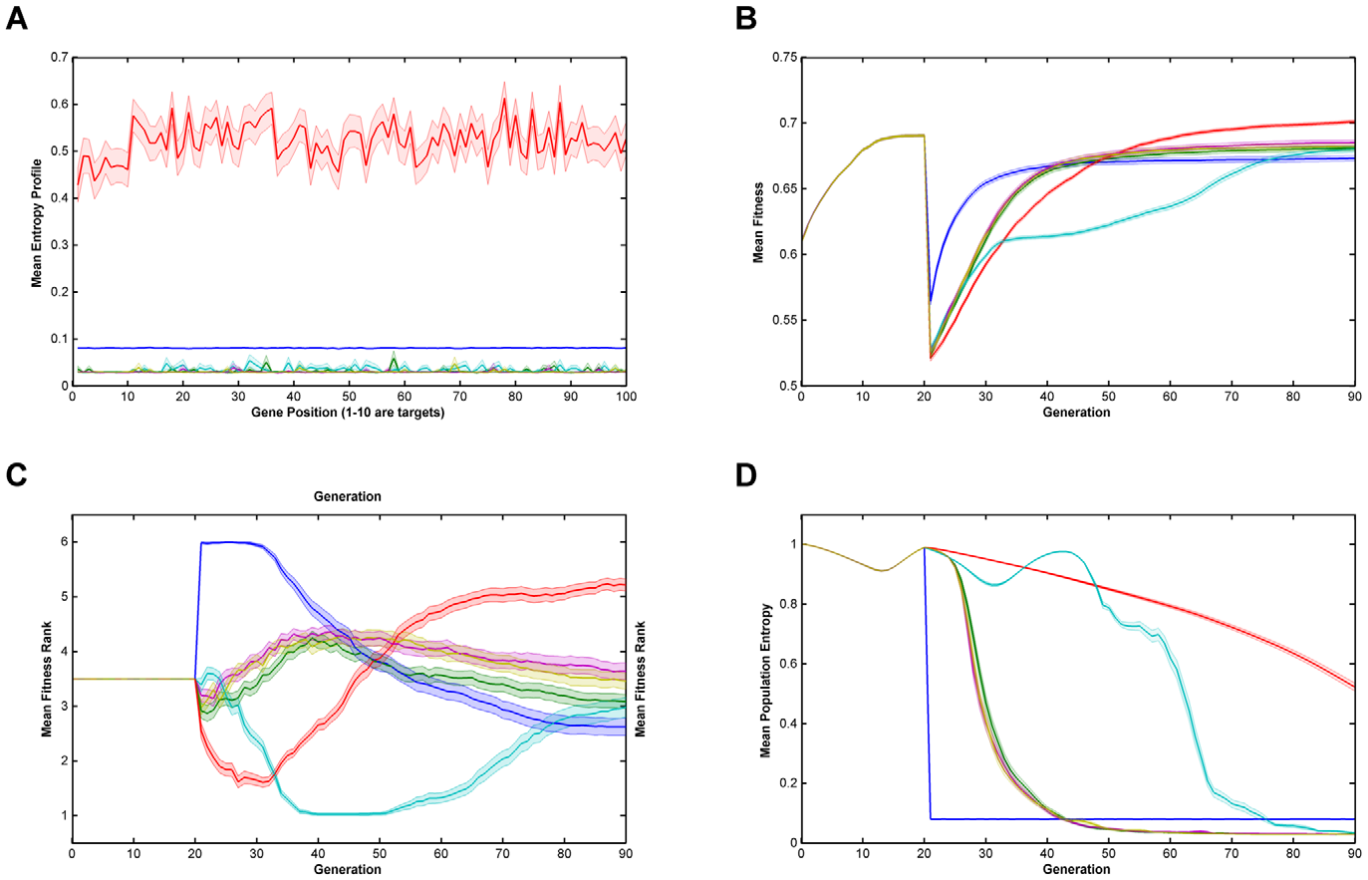
Relative performance of the six algorithms on the modified *NK*-landscapes where λ* = *10000, N = 100, K = 5, r = 10 & burn-in = 20. Note that there a discontinuity in fitness where the algorithm switches from ‘global fitness’, F, to trait fitness, F'. **Key**: **A**. Mean Entropy Profile at Final Generation; **B**. Mean Fitness; **C**. Mean Fitness Rank; **D**. Mean Entropy; —— Breeder; —— Standard GA; —— Local Mating; —— Niching; —— EARL1; EARL2; Error bands are ±1 standard error  = ± stdev/√n.

**Figure 4:**
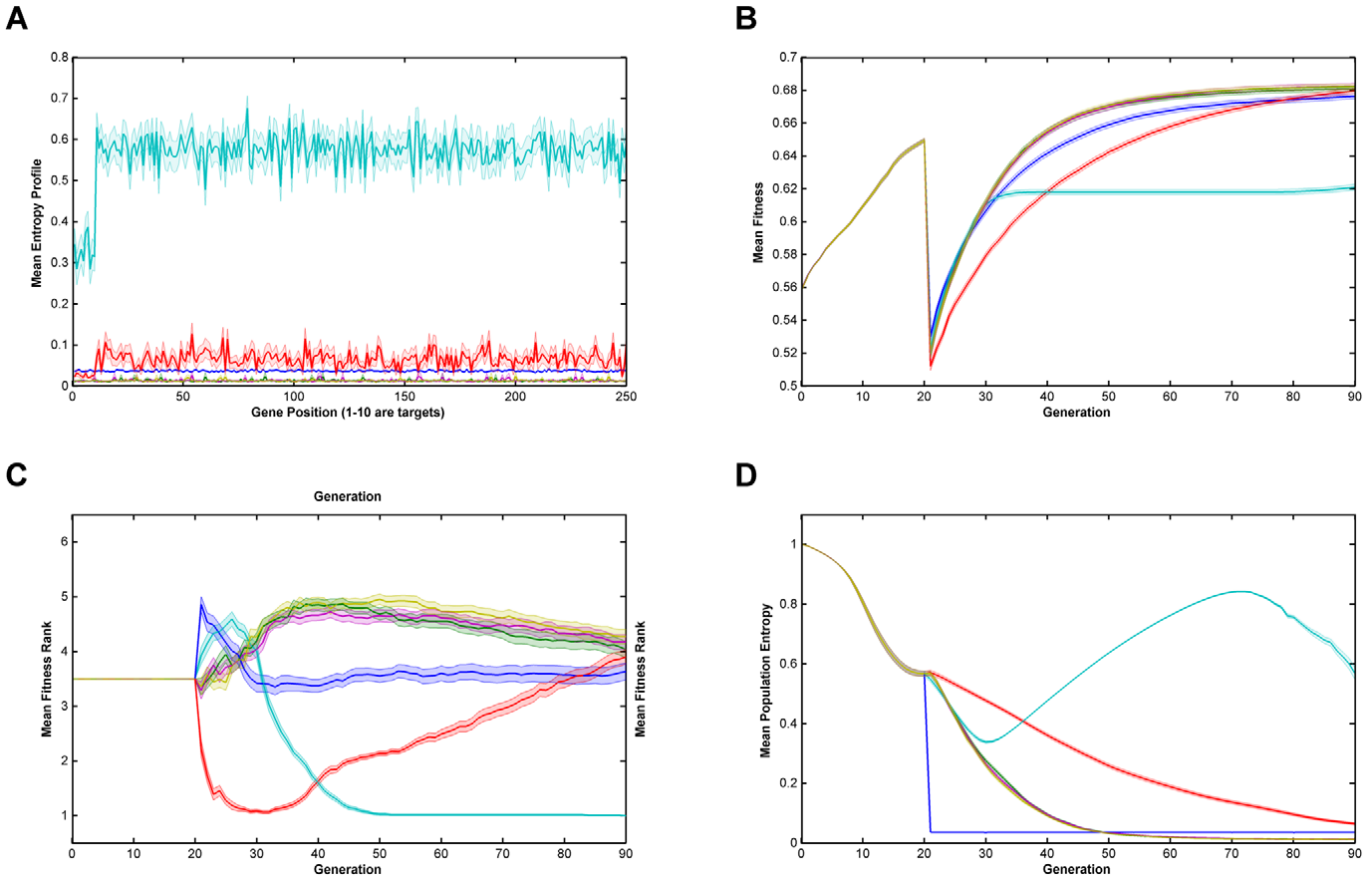
Relative performance of the six algorithms on the modified *NK*-landscapes where λ* = *1000, N = 250, K = 5, r = 10 & burn-in = 20. Note that there a discontinuity in fitness where the algorithm switches from ‘global fitness’, F, to trait fitness, F'. **Key**: **A**. Mean Entropy Profile at Final Generation; **B**. Mean Fitness; **C**. Mean Fitness Rank; **D**. Mean Entropy; —— Breeder; —— Standard GA; —— Local Mating; —— Niching; —— EARL1; EARL2; Error bands are ±1 standard error  = ± stdev/√n.

**Figure 5:**
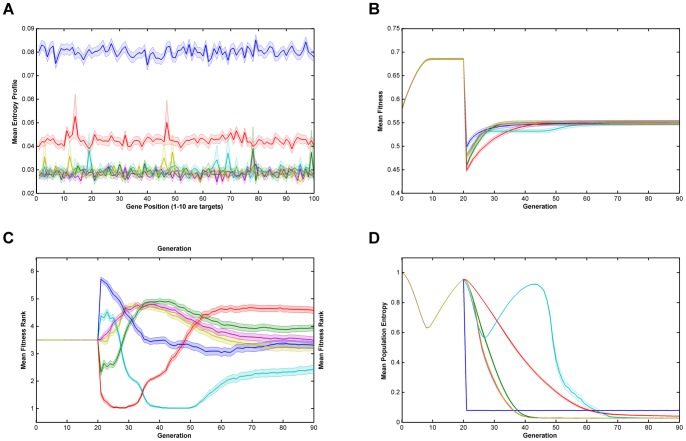
Relative performance of the six algorithms on the modified NK-landscapes where λ* = *1000, N = 100, K = 1, r = 10 & burn-in = 20. Note that there a discontinuity in fitness where the algorithm switches from ‘global fitness’, F, to trait fitness, F'. **Key**: **A**. Mean Entropy Profile at Final Generation; **B**. Mean Fitness; **C**. Mean Fitness Rank; **D**. Mean Entropy; —— Breeder; —— Standard GA; —— Local Mating; —— Niching; —— EARL1; EARL2; Error bands are ±1 standard error  = ± stdev/√n.

There is also an assumption that, given our ability to generate knowledge of genetic sequences (and markers) at massively increasing rates (e.g. [36], [37]), knowledge of parental genotypes should somehow better inform the way in which we carry out molecular breeding programmes, and there is some highly encouraging evidence for this based on ‘genomic selection’ [38]–[46]. Certainly there is increasing interest in and use of marker-assisted selection (MAS) in experimental breeding programmes generally (e.g. (Whittaker et al. 1995; Jones et al. 1997; Moreau et al. 2000; Kim and Park 2001; Dekkers and Hospital 2002; Koebner and Summers 2003; Dekkers 2004; Frary et al. 2005; Williams 2005; Xu et al. 2005; Eathington et al. 2007; Ribaut and Ragot 2007; Collard and Mackill 2008; Hospital 2009; Utomo and Linscombe 2009; Edwards and Batley 2010; Graham et al. 2010; Bancroft et al. 2011)), with particular interest lying in the use of dense genetic markers where available. Again, however, we know of very few published studies [4], [47]–[49] in which the purpose was to compare the effectiveness of breeding programmes that have been performed with and without the use of genetic markers. This matters, because the continuing increase in sequencing speeds means that it is reasonable that before long we shall have the opportunity to acquire complete genome sequences of every organism in a breeding population [50], i.e. knowledge of precisely where we are in the genetic landscape for each organism. It is reasonable that the much lowered costs of sequencing will be outweighed significantly by the knowledge that they bring. However, knowing how much we can benefit from this knowledge, and in particular how best to exploit it (in terms of the breeding algorithms), will be extremely important to the future of plant and animal breeding.

### *In silico* evolution – evolutionary computing

The large field of *in silico* evolution or evolutionary computing has remained surprisingly divorced from ‘real’ biological genetics (albeit that it has been of considerable value in helping our thinking about these landscapes, e.g. [51], [52]; Stadler, 2002; Jones, 1995; Weinberger 1990). Meanwhile, it has developed a plethora of algorithms to search these fitness landscapes with great facility (e.g. [53]–[63]). Since the search spaces are typically astronomic, and the problems NP-hard [64], [65], they seek (as heuristic methods (Pearl, 1984; [55], [66], [67], to find ‘good’ but not provably (globally) optimal solutions.

Evolutionary computing algorithms are based loosely on the principles of Darwinian evolution, involving the generation and analysis of variation, and selection on the basis of one or more measured fitnesses. Typically they are used to solve combinatorial optimisation problems (e.g. [55]) as well as nonconvex continuous problems such as parameter estimation [68] in which there are many possible configurations. The ‘search space’ is the number of possible solutions given the dimensionality of the problem as defined. If there are n variables (dimensions), each of which can take m distinguishable values, the number of possible combinations is evidently m^n^, i.e. such problems scale exponentially with the number of variables. While evolutionary algorithms come in many flavours, some of which we discuss below, in all cases they involve a population of (*in silico*) individuals each encoding a candidate solution to a problem of interest. Each of the members in the population has a fitness that is evaluated. Selection biased by fitness is used to determine which members of the population breed in a given ‘generation’, and offspring of these ‘parent’ individuals are created either by sexual recombination (after matching up of parents), or asexually by cloning a single parent. In either case, mutation is typically applied so that offspring differ from their parents. New individuals are evaluated for fitness, and then these replace (all or some) members of the parent population to form the next generation population. Evolution continues with rounds of mutation/recombination and selection until a desired endpoint is achieved (or a certain number of evaluations performed).

In Genetic Algorithms, both cloning and recombination are biased toward more fit individuals via the tournament selection used; however cloning tends to preserve the population genetic profile, whilst recombination creates more diversity. The (low) mutation rate on both cloned and recombined offspring provides more diversity and helps to prevents stagnation.

In the context of breeding, ‘cloning’, could represent the individual being retained for the study rather than culled; for plant studies ‘cloning’ may be reproduction via cuttings etc. It may be argued that the stochastic nature of the algorithms will mimic the real-world environmental factors that influence breeding experiments.

Much of the literature of evolutionary algorithms is concerned with optimising the nature of the search, typically couched in terms of a tension between ‘exploration’ (wide search to find promising areas of the search space) and ‘exploitation’ (a narrower search to optimise within such areas), while seeking to avoid ‘premature convergence’ to a local optimum that may be far less good than the global (or other local) optimum.

For present purposes, the methods (algorithms) fall into two broad camps: those that at each generation know only the fitnesses of individuals, and those that also know where they are in the sequence/search space (and that thus at each generation increase our knowledge of the landscape). We shall refer to these as Fitness-only (F-) and Genomic (G-) methods. The first question then arises as to whether the kinds of algorithm available with G-methods typically outperform those available with F-methods, in other words whether the genomic knowledge buys you anything, and if so how best to exploit it. A second motivation for the present work hinges on the fact that while the experimental breeding programmes are comparatively slow and costly, computational power is increasingly cheap, and should be exploited (as in projects such as the Robot Scientist [69]–[71], Robot Chromatographer [72] and experimental directed evolution [18]) to optimise the experimental design via ‘active learning’ (see also [73]). Indeed, one algorithm – known as the breeder genetic algorithm [74] – is based on the principles of classical experimental breeding and simply selects the fittest n individuals of a population and breeds with them. Clearly this is likely to lead to premature convergence to a local optimum that is much less fit than others possible. A final recognition is that while no algorithm is best for all landscapes [75] (there is ‘no free lunch’ [76], [77]), some algorithms do indeed do considerably better in specific domains, especially if given some knowledge of the landscape. Indeed, some G-algorithms (e.g. [78]–[81]) explicitly include iterative modelling (‘metamodelling’ [82]–[85]) of the landscapes themselves.

While much of the evolutionary computing literature has assumed access to large populations and generation numbers compared to the smaller populations and 70 generations we used, some methods have purposely focussed on small ones [86], [87], and from the other side we have measured (experimentally) genotype-fitness data on more than 1 million samples (all 10-mers of nucleic acid aptamers [88]). Our discussion does therefore recognise the requirement to keep population sizes reasonably compatible with those likely to be accessible to experimenters.

The chief purposes of the present analysis, then, are (i) to bring to the attention of experimentalists interested in experimental breeding a knowledge of the literature of evolutionary computing, and (ii) to perform a study *in silico* to assess explicitly the kinds of benefits that can be had using knowledge of the genotype relative to those where only the (fitness of) the phenotype is known.

Table 3 illustrates possible correspondences between GA tuneable parameters and a real-world breeding scenario.

We have chosen uniform cross-over [89] in our genetic algorithms rather than tried to simulate real biology more closely. Whilst uniform crossover is reasonably efficient, the disadvantage is that it tends to preserve shorter schemata – however this is offset in that that it may be likely to explore the search-space more thoroughly. We believe this to be a more important consideration given the vast search-space in real genetics. We see no good reason to use other crossover methods since any simulated crossover process that ‘breaks’ a conserved region of coupled genes will not influence the population as the corresponding individual will be automatically culled. In some sense, this means that the algorithm will ‘learn’ the real genetics.

## Experimental

### F- and G-algorithms

As a prelude to comparing F- and G-algorithms on the same landscapes, we here survey a selection of known algorithms of the two types. Tables 1 and 2 therefore summarises a number of the algorithms that have been proposed in the *in silico* literature, distinguishing those (F-algorithms) that know only the fitness (based on the ‘phenotype’) at each generation from those (G-algorithms) that also exploit knowledge of the position in the search space (the ‘genotype’).

There are essentially three ways in which an F-algorithm may be designed to improve over the basic Breeder Genetic Algorithm: (i) by better preserving diversity for longer, with the purpose of preventing convergence to a population that is located on a low fitness peak, and which has little ability to evolve further; (ii) by having mechanisms for tuning its own parameters adaptively, particularly the mutation and crossover rates; and, (iii) by using adaptive walks very near to the fitter ‘parent’ individuals to exploit these solutions more intensively (see Table 1; hybrid or memetic algorithms). Diversity preservation is by far the most important of these mechanisms in our case since (ii) and (iii) will usually demand large numbers of generations to be effective, whereas we are concerned with small generation numbers (see below for why). Although it is probable that most existing experimental breeding programmes do not in fact optimise (i), (ii) and (iii), we felt (a) that we should draw the attention of experimental breeders to this knowledge, and (b) we should not artificially ‘load the dice’ in favour of G-algorithms by using a poor F-algorithm as comparator. Thus we are likely to minimise the perceived benefits of G-algorithms over present practice.

G-algorithms may be able to achieve even more effective diversity preservation, as they have access to far more information, but the question is open. Moreover, G-algorithms have access to a further route to improvement not available to the F-algorithms: they may ‘learn’ or induce explicit statistical models of the sequence-fitness landscape, using this to select for breeding more accurately (see Learnable Evolution Model, metamodel-assisted EAs and EGO in Table 2, and the approach and data in [18]). It is also possible that G-algorithms could be designed to exploit the knowledge of the genotype-fitness map to effect *directed variation* (i.e. mutation and crossover) of individuals, not just to aid in more accurate selection for breeding. However, we exclude this avenue here as it assumes the ability to manipulate specific genes, which is an additional requirement separate from the ability to sequence (know about) the genotypes of individuals (though synthetic biology aims to make this a real possibility in future).

In our experimental study, we cannot hope to show a definitive advantage of one set of algorithms over the other on all problems (in fact that is a doomed proposition due to the No Free Lunch Theorem [75], [76]). Our aims are more illustrative. One aim is to compare the effectiveness (in terms of fitness improvement) of attempts to preserve diversity in an F-algorithm and a G-algorithm. The G-algorithm can explicitly measure and control genetic diversity, whereas an F-algorithm promotes diversity only by restricting mating. A second aim is to assess the relative effectiveness of G-algorithms that learn to model and exploit the fitness-sequence landscape, an approach borrowing from LEM (Table 2). These aims are reflected in the six algorithms that we use in our experiments, described in detail below.

The landscapes on which we evaluate these algorithms are described next.

### Landscapes

The choice of landscapes is more difficult, in that it is known that algorithms can be ‘tuned’ for specific landscapes. However, in this case we are purposely comparing different classes of algorithm on the same landscapes, and we choose landscapes of different character. The character of these landscapes is hard to define exactly, but here the concept of ‘ruggedness’ is important [52], [90]. Ruggedness describes the likelihood that the fitness is well (‘smooth’) or poorly (‘rugged’) correlated with the genotypic distance from a particular starting point. In a very smooth landscape the fitness will decrease smoothly with distance, and the correlation will be good, whereas in a rugged landscape with many peaks and valleys the fitness-distance plot will be much more stochastic and the correlation lower.

Because of the nonlinearity of enzyme kinetics, and the existence of feedback loops, real biochemical networks are highly nonlinear and epistatic (even if they occasionally appear linear/additive for small changes). Thus it is commonly the case that a change in one enzyme A or another enzyme B alone has little effect on a pathway (this follows from the systems properties of networks [91]–[93] and the evolution of biological robustness), but that changes in both have a major effect. This is then epistasis or synergy, and is very well established in both genetics and pharmacology (e.g. [94]–[108]). Epistasis is even observed within individual proteins (e.g. [109]–[115]. What landscapes should we then choose to model?

Despite the increasing availability of ‘genome-scale’ biochemical networks (e.g. [116], [117], these are mainly topological rather than kinetic and so it is not yet possible to model the detailed effects of multiple modulations on ‘real’ (*in silico*) biochemical networks, albeit that there are excellent examples where such models have proved useful in biotechnological optimisation [28], [118]–[121]. For this reason, we have chosen to develop our analyses using ‘artificial’ landscapes with more or less known properties as a guide to the effectiveness (or otherwise) of adding knowledge of the genotype to the knowledge of the phenotype (fitness) during experimental optimisation/breeding programmes.

Whilst not directly modelled after “real” genetics, we believe that the chromosome representation used together with the NK-landscape-based fitness function mimic all the salient features of real genetics, this model is used for both F- and G-algorithms. Whether the algorithm is F- or G-depends on the information obtained (phenotype/genetic information) and selection method used; these are entirely under the control of the experimenter in real breeding programs, therefore we expect no mechanism for unintentional bias towards F-algorithms, since selection is not coupled to the biological model.

### Modified *NK*-landscape

So-called *NK*-landscapes were developed by Kauffman [51], [122]. *NK*-landscapes are a class of synthetic landscapes that describe the epistatic relationships between genes in an organism and the consequent fitness of that organism. In the model genes are represented as bits in a binary string of length *N*, The total fitness *F* of a string (chromosome) is derived from the average fitness contribution of all the positions (genes):
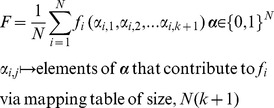



A chromosome, ***α***, is a binary vector of length *N*, ***α*** *∈ {0,1}^Ν^*. The fitness, *f_i_*, of ‘each position’, *i*, is a function of a sub-vector of length *k+1*, which is constructed from a subset of elements drawn from ***α*** such that *α_i,1_  =  α_i_* and *α_i,j_ (j≠i)* are selected uniformly at random without replacement from the elements of ***α***. Thus *f_i_* has *2^k+1^* states, and these are allocated at random in the range *0*→*1*, *f_i_ ∈ [0,1]*.

In the notation *α_i,j_*, ‘*i,j*’ contains the pseudo-subscript ‘*j*’ which represents position along the sub-vector of length *(k+1)*. The mapping of elements of the sub-vector to elements of ***α*** is achieved via a look-up table of size *N(K+1)*.

In NK models, the *N* parameter controls the length of the chromosome and the corresponding search space, and the *K* parameter determines the degree of epistasis. It is not practical computationally to have *N* approach the number of genes found in organisms (yeast: ∼6000, man: ∼24000), so one must choose a suitable compromise that nevertheless gives a sufficiently large search space to be comparable with real breeding populations, we have chosen: *N* = 1000→2^1000^≈10^300^, *N* = 10000→2^10000^≈2×10^3010^. The fitness of a gene depends on *K* other genes; increasing *K* increases the ruggedness of the landscape, for *K* = 0, the fitness function generally has a trivial global optimum that is easy to locate. We have looked at *K* values in line with typical epistasis seen in real organisms.

*NK*-landscapes have been used extensively in the evolutionary computation literature as proxies for a variety of complex systems. It is rare that the exact properties of real biological fitness landscapes are known; however we can modify the properties of our models to the system we are studying more accurately, and the recent empirical studies on DNA binding support the view that *NK* landscapes make a reasonable approximation to real biology [85], [123].

Here, our aim is to use *NK*-landscapes to study the particular set of conditions faced by a human breeder trying to improve certain (quantifiable) traits. We do this by making a small modification to the basic *NK* model. In plants, as in animals and bacteria, certain genes will contribute more strongly to a particular trait than do others. When breeding plants, the aim is to optimize these traits whilst not comprising other qualities of the organism. In *NK*-landscapes the fitness contribution to each bit from all combination of *K*+1 bits is assigned randomly. At low values of *K* the maximum contribution of individual bits may vary greatly across *N*, however as *K* increases this proportionality may be minimized. In modified *NK*-model we weight a portion of the chromosome (*r* bits) more highly by assessing these bits contribution to the overall fitness of the string.
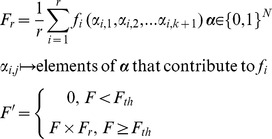



*F*_r_ is multiplied by *F* to generate a new fitness value *F*'. If *F* has a value below the fitness threshold, *F*_th_, *F*' is reduced to 0; this is to simulate the effect of optimizing a trait at the expense of the overall fitness of the organism, and the catastrophic effect of certain deleterious mutations. The fitness threshold, *F*_th_, was set at the minimum value (0.55) for which at least some fraction of the initial population had non-zero *F*'.

## Assessment of Algorithms

Despite the wealth of EAs available our choices are constrained by the nature of the breeding problem. While advances in synthetic biology (e.g. [124]–[126]) may in time lead to the routine synthesis of bespoke genomes, this approach is currently not feasible. Many G algorithms (EDAs, LEM, proSAR) would require the complete synthesis of new chromosomes and so cannot be considered in this study.

We assess the performance of 6 algorithms on a group of modified *NK*-landscapes.

3 F algorithms:

◯ Breeder algorithm (1+λ).
◯ Standard genetic algorithm.
◯ Local mating algorithm.

3 G algorithms:

- ◯ Niching genetic algorithm.
- ◯ Evolutionary algorithm with Rule-based Learning, type 1 (EARL1).
- ◯ Evolutionary algorithm with Rule-based Learning, type 2 (EARL2),

each of which we now describe. As noted, we have sought to tune each beforehand (via preliminary experiments that sought to optimise the mutation, crossover and selection pressure based on tournament size), so as not to give any one an obviously unfair advantage. In the Spirit of Open Science [127], the code is made freely available.

### Breeder algorithm (1+λ)

The breeder algorithm (Mühlenbein and Schlierkamp-Voosen 1993b) is an evolutionary algorithm that uses truncation selection, i.e. the best T% of the offspring population is selected deterministically to become the parents of the next generation. In our version, we use a so-called (1+λ) reproduction scheme: the offspring of a generation all have the same single parent, which is the fittest individual of the previous generation. The offspring are generated by cloning and mutation only. The algorithm has two free parameters, λ, the population size, and *p_m_*, the per-bit mutation rate. The settings for both of these can be seen in Table 4. The algorithm represents a greedy (or strongly elitist) approach that is *a priori* unlikely to be competitive over larger numbers of generations, but may be expected to raise the population mean fitness very quickly. The initialization of the first-generation population used in this and all subsequent algorithms is the same, and is based on the niching GA described below. The procedure is detailed in a separate section below. Like all the algorithms described here, the algorithm terminates when a fixed, pre-ordained number of generations has been reached.

### Standard GA

The standard GA takes the form of a *generational* genetic algorithm, as described in [128]. The population size is λ. Each generation, λ offspring individuals are produced, and these entirely replace the previous generation (even if less fit than the parents, i.e. the algorithm is non-elitist). Let P denote the current population and *P*' denote the population of the next generation. To produce one offspring individual for *P*', a random variate is drawn to determine if the offspring is created by recombination of two parents, followed by mutation, or by cloning followed by mutation. The former occurs with probability *p_c_*, the latter with probability 1-*p_c_*. For offspring produced by recombination, two parents are selected from *P*, with replacement, using *tournament selection* [53], [59] with tournament size = 10 (based on preliminary experiments that suggested that this was optimal). Uniform crossover [89] is used. The resulting individual then undergoes mutation with a per-bit (i.e. per base) mutation rate of p*_m_*. For an individual produced by cloning, just one parent is selected by tournament selection (from *P* with replacement), it is cloned, and the clone undergoes mutation with the same per-bit mutation rate of *p_m_*.

### Local mating

The local mating algorithm follows principles set forth in [129]. The idea is for mating to occur only between individuals that are ‘geographically’ proximal to each other. Specifically, this structured population resists rapid take-over, preserving diversity for longer after a beneficial mutation or recombination event occurs in the population. Our standard generational GA is adapted so that each individual assumes a fixed location on a two-dimensional grid. To construct the next-generation population P', each individual *i* in the current population *P* is taken in turn. If recombination of individual *i* occurs, *i* is recombined with the individual selected by an adapted tournament selection (described below) and the resulting offspring will replace *i* in the next generation population. If only mutation occurs, an individual found by the adapted tournament selection is cloned and mutated, and this will replace *i*. The tournament selection method is adapted so that given an individual *i*, the tournament returns the fittest individual from among the *t_size* ( = 10) neighbours sampled at random (with replacement) from the 24 grid cells that surround *i* in a 5×5 square.

### Niching Genetic Algorithm

The niching GA reduces the fitness of individuals that share a similar genotype with other individuals in the population, a concept known as genotypic fitness sharing [130]. The GA follows the standard one, except that the fitness associated with an individual in the population, which by definition determines its chances for reproduction via the tournament selection, is its shared fitness. The shared fitness is computed over the population by first counting, for each individual, the number of other individuals whose genotypes differ in ‘n_r_’ (the niche radius) or fewer genes; this number is referred to as the niche count *n_c_*. The shared fitness is then *f_share_*  = *10f*/(9+*n_c_*), where *f* is the normal fitness of the individual before sharing is applied (in our version of the algorithm, we have scaled *f_share_* so that if the niche count is one, *f_share_* = *f*.

Simple versions of niching GAs use a fixed and arbitrary value of the niche radius, n_r_; we have adopted a dynamic niche radius scheme whereby the effective niche radius is adjusted at each iteration (generation) of the loop so as to approach a target number of niches, *T_q_*. We estimate the number of niches, *q*, by use of a sampling strategy rather than evaluate the whole population, as the later would be computationally expensive **O**(n^2^). We chose *T_q_* = 5 based on preliminary runs of the niching GA.

### EARL1

Evolutionary Algorithm with Rule-based Learning (EARL1): Devised in the spirit of Michalski's LEM algorithm [131] and Llorà and Goldberg's “wise breeding” algorithm [132], EARL1 uses the statistical model AQ21 [133] which performs pattern discovery, generating inductive hypotheses. This is achieved by employing Attributional Calculus (http://www.mli.gmu.edu/papers/2003-2004/mli04-2.pdf), and produces attributional rules that capture strong regularities in the data, but may not be fully consistent or complete with regard to the training data (it is a ‘fuzzy’ algorithm). Each generation the AQ21 model is trained by selecting the top 20% (T-set) and bottom 20% (L-set) of individuals (in terms of fitness) from the current population. The AQ21 algorithm generates a vector of rules *R* (in the form attributes (loci) and attribute values), which are satisfied in the *H* set but not the *L* set.

When generating a new individual, a rule *r_x_* is selected from *R*, as in LEM the rule is selected weighted by its abundance within the top *T* individuals within the population. Individuals from the current generation are evaluated in terms of whether they satisfy *r_x_*, this subset, *s,* of individuals form a pool from which parents are selected.

Like a standard genetic algorithm, tournament selection (tournament size of 10) is used to select a parent; however, this parent can only be selected from *s*. When recombination is applied, tournament selection is used to select the second parent again from the subset of individuals that obey *r_x_*; *s*. The strategy aims to maintain rules within the next population and prevent the potentially destructive effects of recombination.

### EARL2

EARL2 presents an alternative hypothesis, that the loci that make up individual rules represent interesting points within the search space from which further improvements can be made. EARL2 has the same structure as EARL1, i.e. each new individual within a population is selected from a subset, s, which obeys a selected rule *r_x_*. However when crossover is applied, the second parent is selected by tournament selection from the remainder of the individuals within the population which do not obey *r_x_*.

This approach attempts to disrupt individual rules and may be particularly suited to highly non-linear landscapes, where moving to areas of higher fitness requires breaking of cooperative interaction between loci.

In both EARL1 and EARL2, where no rules are found by the AQ21 algorithm, selection defaults to tournament selection.

### Initialization Procedure (all algorithms)

In crop breeding (and in evolution generally), mutation rates are low [134] and beneficial mutations are rare, arguably reflecting the fact that plants have been evolving for many generations. The standard practice when using NK-landscapes is to generate the binary strings randomly in the first generation of an evolution. Mutating these strings will generate a substantial portion of beneficial mutations, which is unrepresentative of real biology systems. To capture the evolution of plants in a crop breeding programme more accurately, we implement some prior evolution before evaluating the performance of the various algorithms.

In order to simulate a ‘burn-in’ period or warm start to the evolution, i.e. to start with a genetically diverse population of moderately fit individuals that mimics the population one would see in a typical crop-breeding trial, we first run the Niching GA (see above) on the original (unmodified) *NK* landscape for a periods of 0, 20 and 50 generations. The resulting populations are then the starting population for each of the six methods described above, i.e. the starting population of each algorithm is paired (or matched) with the others. This procedure is repeated for each independently drawn *NK* landscape a total of 100 times, giving us 100 matched samples. We ran the algorithms for a further 70 generations after ‘burn-in’.

### Evaluation of algorithms

In addition to plotting the mean fitness and mean population entropy of the repeated runs, we plotted the performance of the algorithms in terms of ranks. In each case (for each setting of N,K,r) we measure the rank of an algorithm, on each of the 100 landscapes, as 1 if it has the lowest NKr fitness score of the C samples and, C if it has the highest fitness, where C is the number of algorithms (here, usually six), other ranks being assigned analogously, with ties being dealt with by assigning the mean rank to each of the tied results in the normal way [135]. For statistical robustness [136] we average these ranks over the 100 landscapes and plot these averages of ranks for each algorithm. This procedure averages the effect of the 100 independent landscapes, and allows us to see easily which algorithms have the best performance, especially where the absolute fitnesses are close together. (One might also argue that such regions indicate convergent performance for all practical purposes).

### Assessment of G-type algorithm performance

In the NKr landscapes, r loci have been artificial weighted in terms of their contribution to overall fitness. Accepting our rationale is correct, EARL1 should identify these r loci and quickly promote convergence on a consensus sequence, while EARL2 will maintain diversity across these positions. The Shannon Entropy *H* [137] is a measure of uncertainty or more precisely the minimum amount of information required to describe a discrete random variable X for a set of K observations x_1_,......,x_k_.
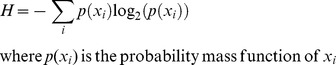



The algorithms were run up to generation 70 on the NKr landscapes beyond the ‘burn-in’ period. Shannon Entropy was measured for each position in the sequence as the mean of one hundred replicates. Convergence on a set of sub-strings across the chromosome will be evidenced by a consequent loss of entropy at these positions and thus demonstrate the mode of action of the two algorithms.

## Results and Discussion

From figures 1, 2, 3, 4 and 5 (and see supplementary information for the raw data), illustrating fitness and entropy, it can be seen the breeder algorithm displays inferior performance relative to the other evaluated algorithms, indicating that strong selection pressure without recombination is not conducive to optimisation on the modified *NK*-landscapes; whilst the fitness (apparently) rises rapidly, the entropy drop almost immediately to near zero, indicating that population diversity is low, conditions typically seen with premature convergence to a local maximum. On aggregate the model-based algorithms (EARL1 and EARL2) display very similar performance to the standard GA. Similarly the Niching algorithm (the other G-type algorithm assessed in this study) is much worse in terms of fitness for most of the early generations, it does however start to converge at generation ∼70 for N = 100; for N = 250 it reaches a plateau. The N parameter determines the gene length, and as the trait fitness is dependent on only 10 loci, any algorithm that preserves overall entropy will have a more detrimental effect on fitness the longer the gene length. Niching does conserver diversity for longer as indicated by the entropy. There appears to be a Diversity/fitness trade-off. The local mating algorithm is the F-type algorithm that also attempts to maintain population diversity; this too exhibits the same kind of trade-off.

The time and expense associated with breeding experiments necessitates the development of an individual with the desired phenotype within the fewest generations possible. The fitness penalty associated with maintaining diversity may therefore be too heavy a price to pay, and any of the EARL1, EARL2 or Standard GA may be preferable – however, EARL1 and EARL2 being more computationally expensive are slower to run an equivalent number of generations, and there appears to be little reason not to favour the simple GA algorithm.

We also looked at results for various values of the ‘ruggedness parameter’, k and the trait bit length, r (k = 1,2,3,5; r = 1,5,10). Whilst there was some inevitable shifting of the positions of the fitness and entropy curves, we did not see any evidence that there was any appreciable change in the relative performance of the algorithms overall. Similarly, runs with low population size (1000) compared to high population size (10000) showed similar behaviour, even though one might expect the higher population to provide a greater reservoir of genetic diversity.

On comparing the trait fitness of runs with no ‘burn-in’ to those with ‘burn-in’ set at 20 and 50 generations, we may gauge the effect of starting from an unfit breeding population compared to ‘well-bred’ population. Burn-in used the niching algorithm so as preserve population diversity. The results indicate that all the algorithms proceed with very similar fitness/entropy profiles (except for the translation of the starting point by virtue of the burn-in period used). Undoubtedly this is due to the trait fitness being very low whether or not any burn-in period is used; in general, the features that contribute to the global fitness will not contribute to the trait fitness chosen by the breeder. Additionally, we found that ‘burn-in’ had already essentially reached a fitness plateau at 20 generations, and that therefore runs for burn-in of 50 did not provide any additional information on algorithm performance.

The successful performance of genetic algorithms in optimisation is widely considered to be derived from their ability to identify a subset of strings with high sequence similarity in certain loci termed schema [128], [138]. In the modified NK-landscapes r loci have been weighted to increase their contribution to the overall fitness of an individual. The ability of the algorithms to identify and optimise these loci will therefore dictate their performance. In figures are displayed the entropy across the loci after the first 70 generations of evolution. From the plots it can be seen the entropy of the first r loci is significantly different depending on the algorithms used and the N parameter chosen. After 70 generations the two entropy-preserving algorithms (niching and local mating) appear to have reduced entropy of the first r loci relative to the remainder of the genome. The remaining algorithms have flat or nearly flat and low entropies. This appears to be an inter-play between the natural trend of genetic algorithms to reduce the entropy in those loci that contain fitness information and the entropy-preserving qualities of the two algorithms. We see little evidence that any of the rule-based algorithms have ‘focused-in’ on relevant loci.

It might seem strange that rule-based approaches would not out-perform simple algorithms such as the Standard GA. Certainly, G-type approaches will still obey the principles of No Free Lunch. For instance it has been previously observed that ProSAR [139] proves effective when addressing landscapes with low levels of epistasis and its bespoke role of optimization of protein sequences in directed evolution experiments, yet performances diminish relative to a standard F-type GA when levels of epistasis are increased [52], [123]. This is arguably unsurprising, given that ProSAR is effectively a piecewise linear algorithm as it is based [140] on partial least squares [141]. Whilst EARL1 and EARL2 should not suffer from the disadvantage of being limited to linear problems (being based on the AQ21 attributional calculus learning module), it is by no means certain that the rule engine employed is suitable to the domain of application; the NKL landscapes we have used may not be amenable to description by such rule sets. In fact, even those runs with k = 1 (low ruggedness/epistasis) showed little or no improvement in the performance of EARL1 and EARL2 algorithms over the standard GA.

These data highlight the gains that can be achieved simply, both through the optimal choice of algorithm and through thoroughly tuning those algorithms to the landscape investigated, consistent with the No Free Lunch theorems alluded to above. We note that the comparative lack of benefit of G-algorithms here contrast somewhat e.g. with that observed in previously problems such as that studied using ParEGO [87]. However, that problem involved many fewer dimension (20) rather than the 1000 considered here, used a greater number of generations (250) than would be feasible in a ‘real’ breeding programme, and involved landscapes that were somewhat less rugged. Given the benefits of combining mutations in multiple known genes for pathway engineering [28], [118]–[121], it seems likely that the greatest benefits of G-algorithms will be manifest when they are able to incorporate prior knowledge.

The benefit of tuning control parameters such as selection pressure and mutation rate is not a new concept to those practised in designing breeding programs. Inbreeding depression is well known as a consequence of over-selection in hermaphroditic crops. Similarly, the gains of increasing mutation rates to increase diversity in evolutionary search have become well known (e.g. [52], [142], [143]). Marker-assisted selection combined with backcrossing is a more direct method of improving phenotypic properties and bears similarities with algorithms that use machine learning to aid the evolutionary processes such as EARL1 and EARL2. The difference here is that we use knowledge of an entire *in silico* genome rather than just a few QTL markers for the breeding programme.

In marker-assisted selection (see above) the marker (usually a QTL, but increasingly a SNP) is used as an indicator of fitness for a desired phenotypic trait. In this approach, the phenotypic trait is assumed to be governed purely by the marker and not through cooperative interactions from elements on distal chromosome locations. The success of MAS is in some part reliant on how well the model reflects reality, and when the phenotypic trait is highly epistatic the model and consequently the evolution will tend to fail. Similarly, the success of evolutionary algorithms augmented with machine learning models will be highly reliant on how well the model reflects the true landscape.

The G-algorithms presented here were designed for applications in experimental breeding programmes, especially when we have sequences for all members of the breeding population of interest. To this end, they would appear to be a highly promising means of understanding the mapping between genotype and phenotype explicitly.

## Concluding Remarks

The increasing availability of genomic (sequence) knowledge, and our need to exploit it in experimental breeding programmes, points up the requirement for exploiting the best algorithms available. Those in use nowadays tend to exploit the methods of statistical genetics, but many more (and different) ones are known in the literature of evolutionary algorithms. We have here surveyed some of these approaches (and note that we do not include any consideration of epigenetics (e.g. [144])), finding that a well-tuned ‘simple’ GA can perform as effectively as some of the more sophisticated rule-based methods in these landscapes, that were not provided with any ‘prior knowledge’ of which genes might enjoy epistatic interactions. This has implications for experimental breeding programmes, especially when we can determine directly what we might wish to produce, for instance using the emerging methods of synthetic biology [126], [145]–[147].

## Supporting Information

File S1 **Raw data for** **Fig 1** **.**
(XLSX)

File S2 **Raw data for** **Fig 2** **.**
(XLSX)

File S3 **Raw data for** **Fig 3** **.**
(XLSX)

File S4 **Raw data for** **Fig 4** **.**
(XLSX)

File S5 **Raw data for** **Fig 5** **.**
(XLSX)

File S6 **zipped file containing non-proprietary code used.**
(ZIP)
